# Effects of Dietary Selenium Sources on Meat Quality, Tissue Selenium Deposition, and Transcriptome Profiling of *Longissimus dorsi* Muscle in Tibetan Pigs

**DOI:** 10.3390/foods15183202

**Published:** 2026-09-10

**Authors:** Xiaobo Guo, Xiaocui Lin, Haopeng Zhong, Daibo Fu, Minfeng Ding, Weican Zhang, Xingping Chen, Xin Li, Tiande Zou, Jun Chen, Jinming You

**Affiliations:** 1 Jiangxi Province Key Laboratory of Animal Nutrition and Feed, Jiangxi Agricultural University, Nanchang 330045, China; guoxiaobo93@126.com (X.G.); zhonghaopeng@stu.jxau.edu.cn (H.Z.); dingminfeng0911@163.com (M.D.); weicanzhang@stu.jxau.edu.cn (W.Z.); cxp0315@jxau.edu.cn (X.C.); 21917011@zju.edu.cn (X.L.); tiandezou@jxau.edu.cn (T.Z.); 2 Ganzhou Animal Husbandry and Fisheries Research Institute, Ganzhou 341000, China; linxiaocui2025@163.com; 3 Jiangxi Research Institute of Selenium-Enriched Agriculture and Industry, Ganzhou 341000, China; 4 Jiujiang Academy of Agriculture Sciences, Jiujiang 332000, China; fudaibo0612@163.com

**Keywords:** *longissimus dorsi muscle*, meat quality, selenium deposition, selenium source, Tibetan pigs, transcriptome profiling

## Abstract

The present study evaluated the effects of dietary selenium sources on growth performance, slaughter traits, meat quality, tissue selenium deposition, and transcriptomic profiles in the *longissimus dorsi* muscle of Tibetan pigs. Seventy-two Tibetan pigs (initial body weight: 41.96 ± 6.91 kg) were randomly allocated to four treatment groups receiving 0.5 mg/kg selenium as either sodium selenite (SeS), selenium-enriched yeast (SeY), selenium-enriched seaweed polysaccharide (SeSP), or nano-selenium (SeN) for 60 days. Results showed that neither growth performance nor slaughter traits differed significantly among the four treatments (*p* > 0.05). Likewise, meat quality parameters of the *longissimus dorsi* remained comparable across groups, with the exception of drip loss, which was significantly increased in the SeN group compared with the SeS, SeY, and SeSP groups (*p* < 0.05), among which no differences were observed (*p* > 0.05). Importantly, selenium contents in the kidney, liver, heart, and *longissimus dorsi* were markedly elevated in the SeY group relative to the other three groups (*p* < 0.05). *Longissimus dorsi* transcriptome analysis identified 34 overlapping differentially expressed genes (DEGs) among the three pairwise comparisons (SeS vs. SeY, SeSP vs. SeY, and SeN vs. SeY), such as *S100A1*, *S100A6*, and *COL22A1* (*p* < 0.05). Functional enrichment analysis revealed that response to external stimulus and extracellular region (GO terms), as well as viral protein interaction with cytokine and cytokine receptor (KEGG pathway), represented shared enriched annotations across all comparisons. In conclusion, selenium-enriched yeast at 0.5 mg/kg proved superior to the alternative selenium sources in promoting selenium bioaccumulation in *longissimus dorsi* of Tibetan pigs. The resultant selenium-enriched muscle tissue exhibited potential beneficial functions including external stimulus response, extracellular region homeostasis, and cytokine–cytokine receptor crosstalk.

## 1. Introduction

Selenium is an essential micronutrient for both animals and humans [1]. Selenium deficiency poses a public health challenge in more than 40 countries and regions around the world, as documented by the World Health Organization [2]. For instance, many residents across Europe, Asia, and Africa consume less than the recommended dietary allowance of 55 µg per day [3]. Selenium-enriched foods are gaining widespread consumer appeal not only for meeting the nutritional needs of selenium-deficient populations, but also for enhancing physiological and immune functions in the general public [4]. The high global consumption of pork, combined with the efficient bioaccumulation of selenium in pork through feed fortification, makes it an effective vehicle for delivering this essential trace element to human populations [5].

Selenium accumulation in pork is directly influenced by dietary supplementation levels in feed. A comparison of international standards reveals that the recommended selenium level for finishing pigs is 0.15 mg/kg according to both NRC (2012) [6] and the Nutritional Requirements of Chinese Pigs (GB/T 39235-2020) [7], but 0.30 mg/kg under ARC (1981) guidelines [8]. In commercial practice, finishing pigs are typically supplemented with 0.3 mg/kg selenium, and the upper limit supplementation level is 0.5 mg/kg in the EU and China [9]. In our previous study, increasing selenium from 0.2 to 0.5 mg/kg markedly increased loin selenium deposition and improved meat quality of finishing pigs [10]. However, studies comparing different selenium sources at this 0.5 mg/kg level regarding selenium deposition and meat quality in finishing pigs are limited.

Dietary selenium source is another factor influencing selenium accumulation in pork. Sodium selenite, an inorganic compound, has been widely used in the swine industry [11]. Selenium-enriched yeast, produced by enriching selenium within the cellular protein structures of growing yeast, represents a major organic selenium source in pig production [12,13]. The main form of selenium in selenium-enriched yeast is selenomethionine [14], which has been demonstrated to have biological functions [15,16]. Selenium-enriched seaweed polysaccharide, extracted from algae, is characterized by selenium bound to organic substances such as polysaccharides and proteins, forming biologically active organic selenium compounds that exhibit lower toxicity and higher bioactivity than inorganic forms [17,18]. Selenium in selenium-enriched seaweed polysaccharide mainly exists as covalently bound selenium-polysaccharide complexes, along with minor amounts of selenoamino acids and inorganic selenium [17,18]. Nano-selenium refers to elemental selenium nanoparticles (1–100 nm) prepared by physical or chemical methods [17,18]. These sources differ markedly in their properties: sodium selenite is the conventional inorganic option; selenium-enriched yeast offers superior bioavailability; nano-selenium demonstrates high absorption efficiency and low toxicity; and selenium-enriched seaweed polysaccharide provides a natural organic alternative.

Chinese indigenous pig breeds are gaining increasing popularity among consumers, and Tibetan pigs are particularly favored among them [19]. Accordingly, we selected Tibetan pigs as the experimental subjects for this study. This study aimed to investigate the effects of dietary supplementation with different selenium sources (sodium selenite, selenium-enriched yeast, selenium-enriched seaweed polysaccharide, and elemental nano-selenium) at 0.5 mg/kg on the growth performance, slaughter traits, meat quality, tissue selenium deposition, and *longissimus dorsi* muscle transcriptome of Tibetan pigs, with the objective of providing a basis for selenium source selection in the production of selenium-enriched pork.

## 2. Materials and Methods

### 2.1. Animal Experimental Design

After a 7-day acclimatization period, 72 healthy Tibetan pigs without lameness and abnormal behavior (36 barrows and 36 gilts; initial body weight: 41.96 ± 6.91 kg; initial age: approximately 5 months) were selected and allocated by body weight and sex to four dietary treatment groups, with six replicate pens per group and three pigs per pen. During the feeding trial, the pigs were reared under environmentally controlled conditions, where each pen (2.0 m × 3.0 m) provided a stocking density of 2.0 m^2^ per pig. The four experimental diets were formulated by supplementing a basal diet with 0.5 mg/kg selenium from different sources: sodium selenite (SeS, 1000 mg selenium/kg; Yuandazhongzheng Bio-technology Co., Ltd., Shijiazhuang, China), selenium-enriched yeast (SeY, 1000 mg selenium/kg; Angel Yeast Co., Ltd., Yichang, China), selenium-enriched seaweed polysaccharide (SeSP, 160 mg selenium/kg; Pengyang Biological Engineering Co., Ltd., Qingdao, China), or nano-selenium (SeN, 2000 mg selenium/kg; Boshiao Biotechnology Co., Ltd., Guangzhou, China). The basal diet (Table 1) was formulated to meet the nutrient requirements recommended by the Chinese National Feed Standard for swine [7], with the exception of selenium. The analyzed selenium content was 0.56, 0.57, 0.57, and 0.56 mg/kg in the SeS, SeY, SeSP, and SeN-supplemented diets, respectively. Pigs were fed the experimental diets for 60 days with ad libitum access to feed and water.

### 2.2. Data and Sample Collection

All pigs were weighed at the beginning and end of the trial. Feed intake was recorded on a per-pen basis throughout the experimental period, and average daily gain (ADG), average daily feed intake (ADFI), and feed conversion ratio (FCR) were subsequently calculated. At the conclusion of the feeding trial, one pig per pen (replicate, six pigs per treatment) with a body weight closest to the pen average was selected for slaughter by exsanguination following electrical stunning. Slaughter procedures followed the protocols described in our previous study [20]. Measurements of carcass weight, body length, body diagonal length, backfat thickness, and *longissimus dorsi* eye area were performed in accordance with the *Technical Specification for Measurement of Carcass Traits in Lean-Type Pigs* (NY/T 825-2004, China) [21]. The *longissimus dorsi* muscle was sampled from the left half-carcass, specifically from the rib section extending from the 10th to the 13th rib.

### 2.3. Proximate Composition of Longissimus Dorsi Muscle

The moisture, crude protein, crude fat, and crude ash contents of the meat samples (*longissimus dorsi* muscle) were determined in accordance with the corresponding national food safety standards of China: GB 5009.3-2016 [22], GB 5009.5-2016 [23], GB 5009.6-2016 [24], and GB 5009.4-2016 [25], respectively.

### 2.4. Meat Quality of Longissimus Dorsi Muscle

The pH, lightness (L*), redness (a*), yellowness (b*), shear force, cooking loss, and drip loss of the *longissimus dorsi* samples were measured according to the methods described in our previous study [20]. Briefly, the pH was measured using a portable pH meter (PH818M, Smart, Hong Kong, China). Meat color, including lightness (L*), redness (a*), and yellowness (b*), was measured three times at different locations of *longissimus dorsi* samples using a colorimeter (3nh Technology Co., Ltd., Shenzhen, China), and the mean of the three measurements was used for statistical analysis. To measure drip loss, *longissimus dorsi* samples (5 cm × 2 cm × 1 cm) were excised and weighed (W1) within two hours of slaughter. Each specimen was then transferred to a drip loss tube and hung at 4 °C for 24 h. Following careful removal of surface moisture, the samples were weighed again (W2). Drip loss was subsequently determined using the formula: Drip loss (%) = (W1 − W2)/W1 × 100. To determine cooking loss, *longissimus dorsi* samples (2.5 cm thick) were weighed (M1), vacuum-sealed, and heated in a water bath at 80 °C until the core temperature reached 75 °C. After cooling, the cooked samples were reweighed (M2), and cooking loss was calculated as follows: Cooking loss (%) = (M1 − M2)/M1 × 100. To measure shear force, cooked *longissimus dorsi* samples were cut into 25 mm × 25 mm strips, with muscle fibers oriented parallel to the longitudinal axis. Shear force was measured perpendicular to the fiber direction using a C-LM3B digital muscle tenderness meter (Tenovo, Beijing, China). Each sample was measured in triplicate, and the mean value was expressed in Newtons (N).

### 2.5. Tissue Selenium Content

The selenium content in the diet, kidney, liver, heart, and *longissimus dorsi* was determined as described in our previous study [10]. In brief, selenium standards, diets, or wet tissue samples were digested with a nitric-perchloric acid mixture to oxidize selenium complexes to Se^4+^. Under acidic conditions, Se^4+^ reacted with 2,3-diaminonaphthalene to form 4,5-benzopiaselenol, which was then extracted with cyclohexane. The organic phase was analyzed for fluorescence intensity using a spectrofluorometer (RF-5301, Shimadzu, Kyoto, Japan) at an excitation wavelength of 376 nm and an emission wavelength of 520 nm. Fluorescence intensity was linearly correlated with selenium concentration at sample Se concentrations below 0.5 μg/g. The limit of detection for this method was 0.01 μg/g.

### 2.6. Transcriptome Analysis of Longissimus Dorsi Muscle

#### 2.6.1. RNA Extraction and Sequencing

Total RNA was isolated from *longissimus dorsi* muscle using Trizol reagent (Invitrogen, Carlsbad, CA, USA), and its integrity, concentration, and purity were assessed by agarose gel electrophoresis and NanoDrop 2000 spectrophotometry (Thermo Scientific, Waltham, MA, USA), respectively. All samples had RNA integrity numbers (RIN) ≥ 7.5, A260/A280 ratios between 1.8 and 2.1, and A260/A230 ratios > 1.8. Sequencing libraries were prepared as reported [26], using the Illumina NovaSeq Reagent Kit (Illumina, San Diego, CA, USA) following mRNA enrichment with oligo(dT) beads. Paired-end sequencing was performed on the NovaSeq 6000 platform (Illumina) at Shanghai Personal Biotechnology Co., Ltd. (Shanghai, China). The average sequencing depth was ~6.0 Gb per sample.

#### 2.6.2. Identification of Differentially Expressed Genes (DEGs)

The raw sequencing data were processed with Fastp (v0.22.0) to filter out low-quality reads and generate clean data. The high-quality reads were subsequently aligned to the pig reference genome (*Sus scrofa domesticus*, Sscrofa11.1, GenBank assembly accession: GCA_000003025.6) using HISAT2 (v2.1.0). The overall mapping rate ranged from 89.2% to 93.5%. Read counts per gene were then calculated with HTSeq (v0.9.1) as the raw expression values, and FPKM was employed for expression normalization. Differential gene expression was analyzed using DESeq2 with the following criteria: |log_2_FoldChange| > 1 and *p*-value < 0.05.

#### 2.6.3. GO and KEGG Pathway Enrichment Analysis

Gene Ontology (GO) and Kyoto Encyclopedia of Genes and Genomes (KEGG) enrichment analysis of differentially expressed genes was performed using topGO (v2.50.0) and ClusterProfiler (v4.6.0), respectively, with *p*-values calculated by the hypergeometric distribution (significance: *p* < 0.05). The top 20 significantly enriched GO terms and KEGG pathways were visualized as scatter plots.

### 2.7. Statistical Analysis

For growth performance parameters (ADFI, ADG, and FCR), the pen was considered the experimental unit. For all other measured parameters, an individual pig selected from each pen for slaughter and sampling was regarded as the experimental unit. Data except for transcriptome data were analyzed by one-way analysis of variance (ANOVA) with SPSS statistical software 22.0 (IBM, Chicago, IL, USA), along with Tukey’s HSD post hoc test for conducting multiple comparisons among treatments. Data were reported as mean values and standard error of the means (SEM); a result with *p* < 0.05 was considered statistically significant.

## 3. Results

### 3.1. Growth and Slaughter Performance

The effects of dietary selenium sources on the growth and slaughter performance of Tibetan pigs are presented in Table 2. Dietary selenium source treatments did not affect the average daily feed intake, average daily gain, and feed conversion ratio of Tibetan pigs (*p* > 0.05). Similarly, slaughter performance parameters, including carcass weight, body length, body diagonal length, backfat thickness, and *longissimus dorsi* area, remained unaffected by the dietary treatments (*p* > 0.05).

### 3.2. Meat Quality

The effects of dietary selenium sources on the meat (*longissimus dorsi*) quality of Tibetan pigs are shown in Table 3. Dietary selenium source treatments did not affect the moisture, crude protein, crude fat, crude ash, cooking loss, shear force, pH, lightness, redness, and yellowness of *longissimus dorsi* from Tibetan pigs (*p* > 0.05). However, drip loss was higher in the SeN group than in the SeS, SeY, and SeSP groups (*p* < 0.05), with no statistical difference among the latter three groups (*p* > 0.05).

### 3.3. Tissue Selenium Deposition

**Figure 1:**
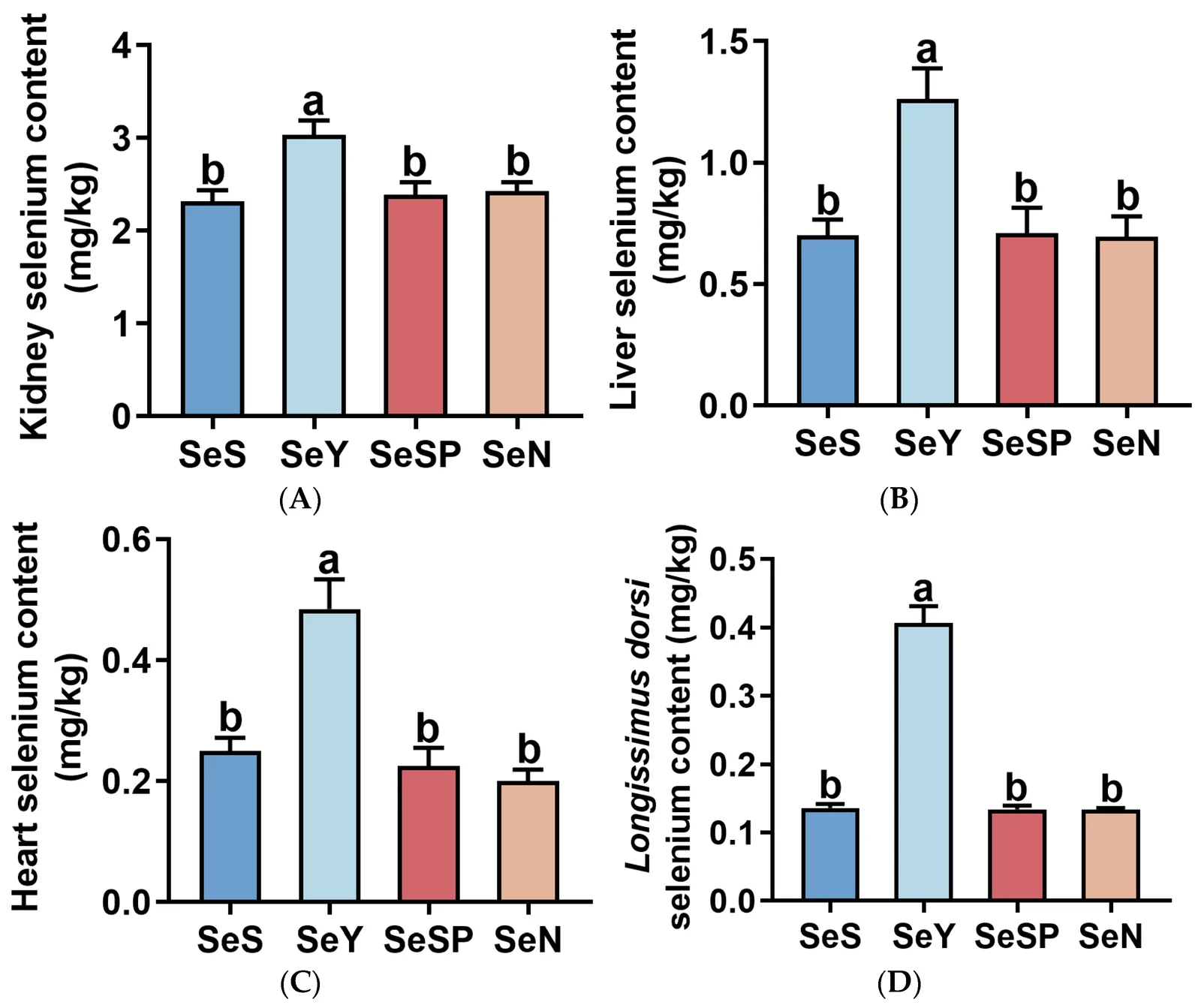
Effects of dietary selenium sources on tissue selenium deposition in Tibetan pigs (*n* = 6, mean ± SEM). (**A**–**D**), Selenium content (mg/kg wet weight) in the kidney, liver, heart, and *longissimus dorsi*, respectively. ^a,b^ Within each panel, values not sharing a common letter differ significantly (*p* < 0.05). Abbreviations: SeS, sodium selenite; SeY, selenium-enriched yeast; SeSP, selenium-enriched seaweed polysaccharide; SeN, nano-selenium.

Effects of dietary selenium sources on tissue selenium deposition in Tibetan pigs are shown in Figure 1. The selenium content was highest in the kidney, followed by the liver, heart, and *longissimus dorsi* of Tibetan pigs. The selenium contents in the kidney, liver, heart, and *longissimus dorsi* of Tibetan pigs were significantly increased in the SeY group compared with the SeS, SeSP, and SeN groups (*p* < 0.05).

### 3.4. Transcriptome Analysis of Longissimus Dorsi

#### 3.4.1. Differential Expression Analysis

**Figure 2:**
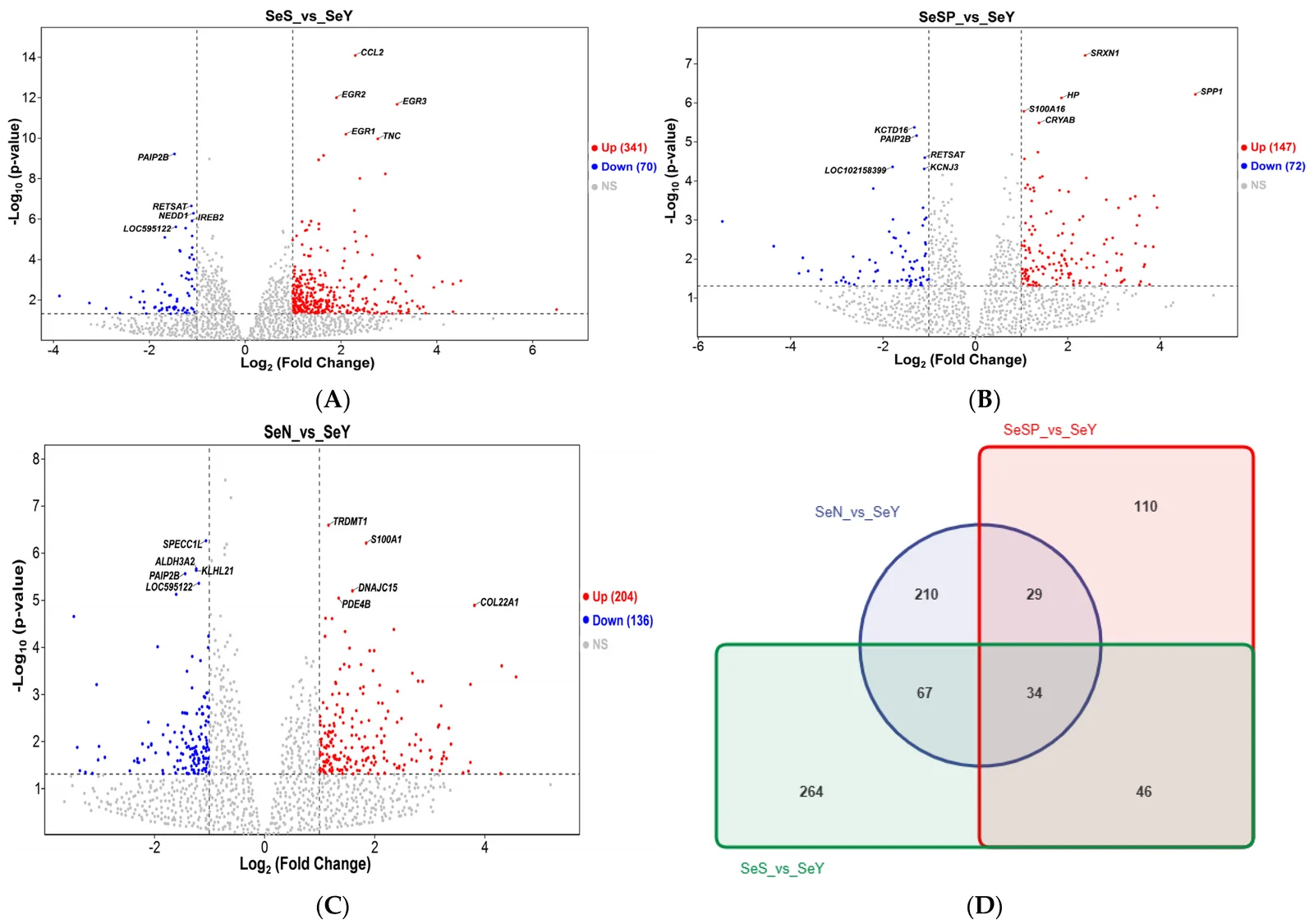
Transcriptome profile of differentially expressed genes (DEGs) in the *longissimus dorsi* of Tibetan pigs (*n* = 6). (**A**–**C**). Volcano plots of DEGs in the *longissimus dorsi* comparing the SeY group with the SeS, SeSP, and SeN groups, respectively. (**D**). Venn diagram of DEGs for SeS_vs_SeY, SeSP_vs_SeY, and SeN_vs_SeY. Abbreviations: SeS, sodium selenite; SeY, selenium-enriched yeast; SeSP, selenium-enriched seaweed polysaccharide; SeN, nano-selenium.

Differentially expressed genes (DEGs) in the *longissimus dorsi* of Tibetan pigs were identified using |log_2_FoldChange| > 1 and *p*-value < 0.05 as thresholds, comparing SeS vs. SeY, SeSP vs. SeY, and SeN vs. SeY. As shown in Figure 2A–C, 411, 219, and 340 DEGs were identified for SeS vs. SeY, SeSP vs. SeY, and SeN vs. SeY, respectively, including 341, 147, and 204 upregulated and 70, 72, and 136 downregulated genes. Specifically, compared with the SeS group, the SeY group exhibited upregulated expression of genes including *CCL2*, *EGR1*, *EGR2*, *EGR3*, and *TNC*, and downregulated expression of genes including *PAIP2B*, *RETSAT*, *NEDD1*, *IREB2*, and *LOC595122* (*p* < 0.05) (Supplementary File S1). Compared to the SeSP group, the SeY group showed upregulated expression of genes such as *SRXN1*, *SPP1*, *HP*, *S100A16*, and *CRYAB*, and downregulated expression of genes such as *KCTD16*, *PAIP2B*, *RETSAT*, *LOC102158399*, and *KCNJ3* (*p* < 0.05) (Supplementary File S2). Relative to the SeN group, the SeY group displayed upregulated expression of genes including *TRDMT1*, *S100A1*, *DNAJC15*, *PDE4B*, and *COL22A1*, and downregulated expression of genes such as *SPECC1L*, *ALDH3A2*, *KLHL21*, *PAIP2B*, and *LOC595122* (*p* < 0.05) (Supplementary File S3).

**Figure 3:**
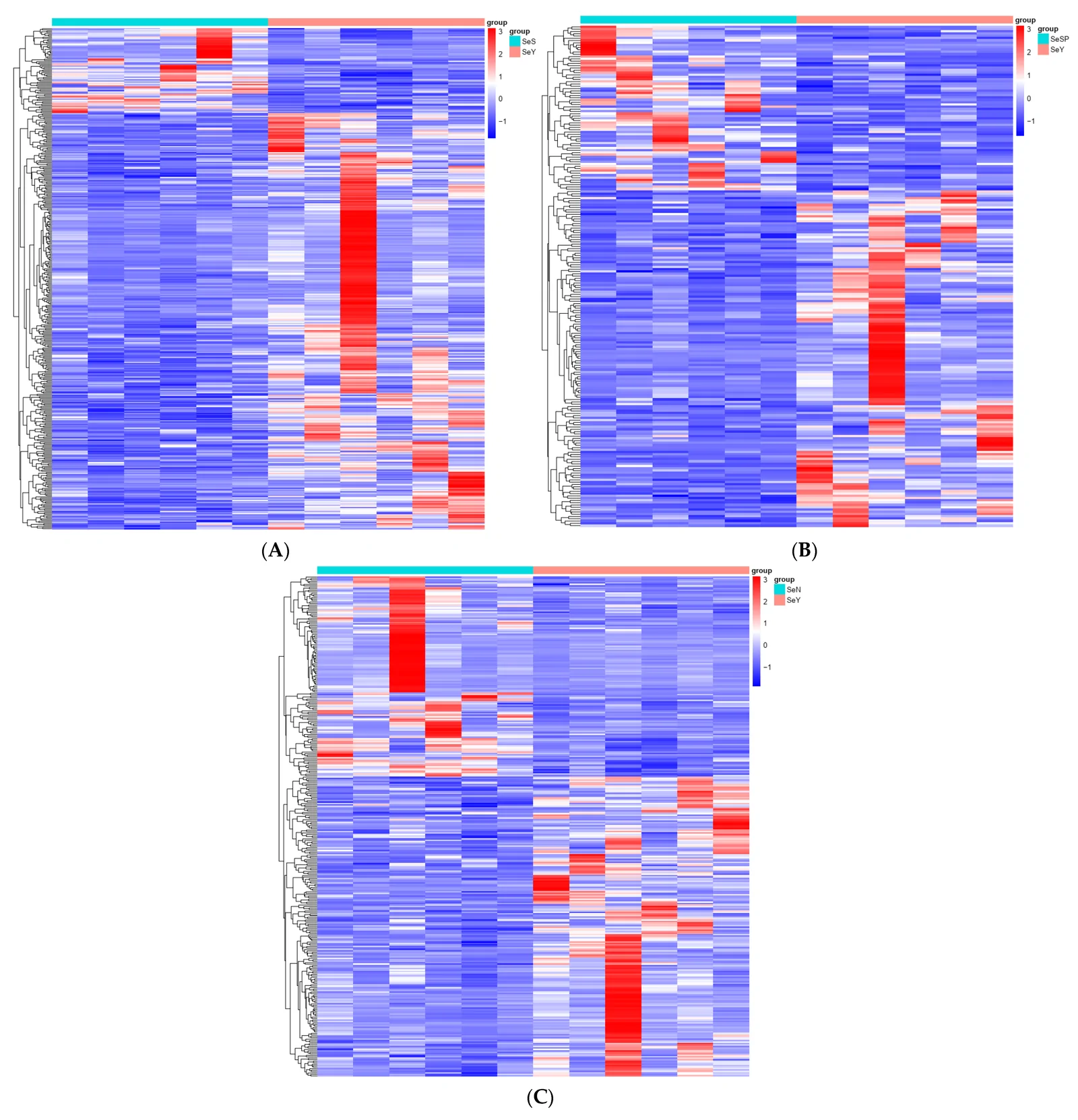
Heatmap of the expression levels of DEGs in the *longissimus dorsi* of Tibetan pigs among treatment groups (*n* = 6). (**A**–**C**). Heatmaps of DEGs in the *longissimus dorsi* comparing the SeY group with the SeS, SeSP, and SeN groups, respectively. Abbreviations: SeS, sodium selenite; SeY, selenium-enriched yeast; SeSP, selenium-enriched seaweed polysaccharide; SeN, nano-selenium.

All DEGs underwent hierarchical clustering to characterize sample–gene relationships based on expression levels, with heatmaps depicting the results (Figure 3A–C). Columns represent individual samples and rows represent genes. A Venn diagram (Figure 2D) was constructed to display the overlaps of DEGs among the comparison groups. A total of 34 DEGs were shared across all three comparisons (SeS vs. SeY, SeSP vs. SeY, and SeN vs. SeY) (Table 4). Specifically, compared with the SeS, SeSP, and SeN groups, the SeY group exhibited upregulated expression of *S100A1*, *S100A6*, *COL22A1*, *TNC*, *PRG4*, *MT1A*, *GPNMB*, *GIMAP4*, *LOC100512920*, *CD200R1*, *FCER1G*, *SCIN*, *FOSL1*, *BAIAP2L1*, *LOC110258709*, *HABP2*, *PRSS55*, *GALP*, *IFI30*, *NECAB3*, *PRSS35*, *UNC13A*, *LOC110261486*, *LOC100516039*, *LOC100521785*, *E2F8*, and *RRH*, and downregulated expression of *PAIP2B*, *RETSAT*, *PTCHD4*, *NFATC3*, *CBL*, *KCNJ3*, and *LOC102161970* (*p* < 0.05).

#### 3.4.2. GO Enrichment Analysis of Differentially Expressed Genes (DEGs)

**Figure 4:**
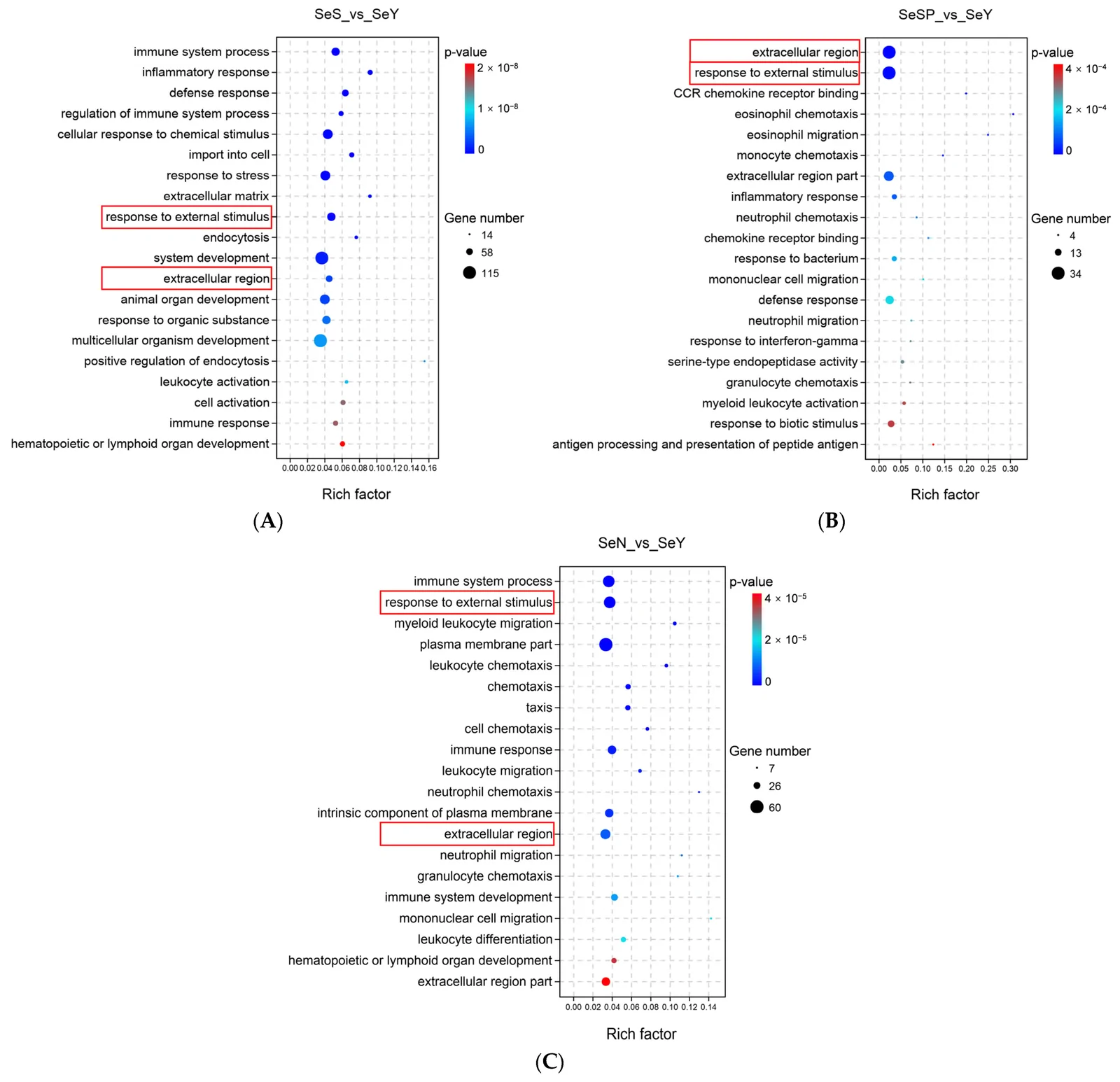
GO enrichment analysis of DEGs in the *longissimus dorsi* of Tibetan pigs among treatment groups (*n* = 6). (**A**–**C**). Top 20 GO pathways of DEGs in the *longissimus dorsi* comparing the SeY group with the SeS, SeSP, and SeN groups, respectively. Abbreviations: SeS, sodium selenite; SeY, selenium-enriched yeast; SeSP, selenium-enriched seaweed polysaccharide; SeN, nano-selenium.

The DEGs were subjected to GO enrichment analysis (Supplementary Files S4–S6). Figure 4A–C presents the top 20 GO terms enriched by DEGs in the *longissimus dorsi* across the three comparisons: SeS vs. SeY, SeSP vs. SeY, and SeN vs. SeY, respectively. The results revealed that in SeS_vs_SeY, the DEGs were primarily enriched in biological processes including the immune system process, inflammatory response, defense response, regulation of immune system process, and cellular response to chemical stimulus. For SeSP_vs_SeY, the DEGs were mainly enriched in extracellular region, response to external stimulus, CCR chemokine receptor binding, eosinophil chemotaxis, and eosinophil migration. For SeN_vs_SeY, the DEGs were predominantly enriched in immune system process, response to external stimulus, myeloid leukocyte migration, plasma membrane part, and leukocyte chemotaxis. Interestingly, response to external stimulus and extracellular region emerged as shared enriched terms among all three comparisons (SeS_vs_SeY, SeSP_vs_SeY, and SeN_vs_SeY).

#### 3.4.3. KEGG Pathway Analysis of Differentially Expressed Genes (DEGs)

**Figure 5:**
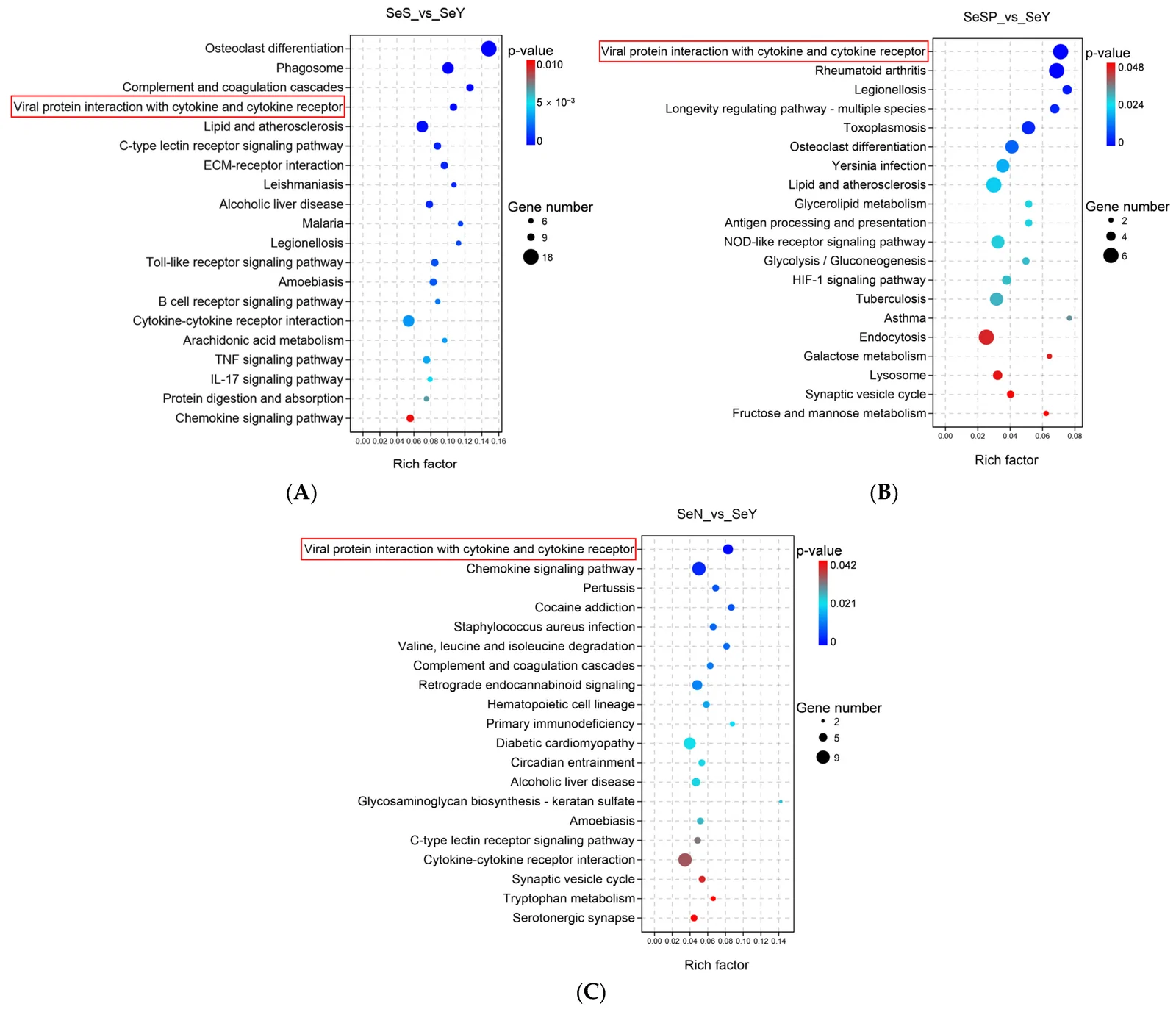
KEGG enrichment analysis of DEGs in the *longissimus dorsi* of Tibetan pigs among treatment groups (*n* = 6). (**A**–**C**). Top 20 KEGG pathways of DEGs in the *longissimus dorsi* comparing the SeY group with the SeS, SeSP, and SeN groups, respectively. Abbreviations: SeS, sodium selenite; SeY, selenium-enriched yeast; SeSP, selenium-enriched seaweed polysaccharide; SeN, nano-selenium.

The DEGs were subjected to KEGG pathway enrichment analysis (Supplementary Files S7–S9). Figure 5A–C displays the top 20 KEGG pathways of DEGs in the *longissimus dorsi* comparing the SeY group with the SeS, SeSP, and SeN groups, respectively. The results revealed that the differentially expressed genes in SeS_vs_SeY were significantly enriched in several pathways, including osteoclast differentiation, phagosome, complement and coagulation cascades, viral protein interaction with cytokine and cytokine receptor, and lipid and atherosclerosis. For SeSP_vs_SeY, the differential genes were significantly enriched in pathways such as viral protein interaction with cytokine and cytokine receptor, rheumatoid arthritis, legionellosis, longevity regulating pathway-multiple species, and toxoplasmosis. For SeN_vs_SeY, significant enrichment was observed in viral protein interaction with cytokine and cytokine receptor, chemokine signaling pathway, pertussis, cocaine addiction, and *Staphylococcus aureus* infection. Of note, the viral protein interaction with cytokine and cytokine receptor was commonly enriched across all three comparisons (SeS_vs_SeY, SeSP_vs_SeY, and SeN_vs_SeY).

## 4. Discussion

This study was the first to investigate the effects of dietary selenium sources at a 0.5 mg/kg supplementation level on meat quality, tissue selenium deposition, and transcriptome profiling of the *longissimus dorsi* muscle in pigs. The results revealed that different selenium sources had no significant impact on average daily feed intake, average daily gain, or feed conversion ratio. These findings align with Tian et al. (2006), who reported that supplementation with 0.5 mg/kg selenium, either as sodium selenite or selenium-enriched yeast, did not alter the growth performance of growing-finishing pigs [27]. Furthermore, our previous research demonstrated that elevating dietary selenium from 0.2 mg/kg to 0.5 mg/kg did not affect growth performance in finishing pigs [10]. These results suggest that 0.5 mg/kg selenium supplementation, irrespective of source, exerts no negative effects on the growth performance of finishing pigs. Our results also indicate that 0.5 mg/kg selenium supplementation, regardless of selenium sources, had no impact on the slaughter traits of Tibetan pigs. Collectively, the present findings demonstrate that 0.5 mg/kg selenium represents a safe and permissible dose that complies with the maximum regulatory limits [9], without compromising growth performance or carcass traits, thereby validating its use in selenium-enriched pork production.

We next evaluated the meat quality of Tibetan pigs fed diets supplemented with different dietary selenium sources. The results showed that dietary selenium source treatments did not affect the moisture, crude protein, crude fat, or crude ash contents of the *longissimus dorsi* muscle. Consistent with our results, Zhan et al. (2007) reported that crude protein and crude fat in the loin of finishing pigs were unaffected by dietary selenium sources (sodium selenite and selenomethionine) [28]. In addition, Gjerlaug-Enger et al. (2014) found that combined dietary supplementation with 0.4 mg/kg selenium and rapeseed products also did not impact muscle moisture, protein, or intramuscular fat contents in pigs [29]. Interestingly, while cooking loss, shear force, pH, lightness, redness, and yellowness of the *longissimus dorsi* muscle remained comparable across all four selenium source groups, drip loss was markedly elevated in the SeN group relative to the SeS, SeY, and SeSP groups, among which no significant difference was detected. This finding is supported by Jing et al. (2024), who demonstrated that nano-selenium provided limited protection against heat stress-induced deterioration of breast muscle meat quality in broilers compared with sodium selenite, selenium-enriched yeast, and selenomethionine [30]. Consistently, these authors observed significantly higher drip loss in the nano-selenium group than in the other three groups [30]. They attributed this weaker protective effect to the inferior regulatory efficiency of nano-selenium in modulating the expression of key selenoproteins in muscle tissue [30].

The selenium contents in the liver, kidney, heart, and *longissimus dorsi* muscle are commonly used indicators reflecting selenium nutritional status and selenium deposition in pigs. Accordingly, we measured selenium deposition in pig tissues in response to dietary selenium sources at a level of 0.5 mg/kg. Selenium content was highest in the kidney, followed by the liver, heart, and *longissimus dorsi* muscle, which is consistent with the findings of Mahan et al. (1999) [31] and Zhang et al. (2020) [32]. The selenium contents in the kidney, liver, heart, and *longissimus dorsi* of Tibetan pigs were significantly higher in the SeY group than in the SeS, SeSP, and SeN groups. This indicates that selenium-enriched yeast exhibited the highest selenium deposition efficiency among the four selenium sources in pigs. In line with our results, Jiang et al. (2017) reported greater selenium deposition in the loin of finishing pigs fed 0.3 mg/kg selenium as selenium-enriched yeast compared with sodium selenite, and a further increase in loin deposition when the dietary selenium level was raised from 0.3 mg/kg to 0.5 mg/kg in the form of selenium-enriched yeast [33]. In broilers, Chen et al. (2024) conducted a similar experimental design with selenium level (0.5 mg/kg) and selenium sources [34]. The authors found that selenium contents in both breast and thigh muscles were markedly higher in the selenium-enriched yeast group than in the other groups, among which no significant differences were detected [34]. These findings further support our results and suggest that selenium derived from dietary selenium-enriched yeast is more efficiently deposited in muscle tissue compared with other selenium sources. Such discrepancies in deposition efficiency may arise from variations in the absorption mechanisms and metabolic pathways of different selenium forms [35,36]. Selenomethionine, which is the main form of selenium in selenium-enriched yeast (organic selenium), can be absorbed via the methionine transport system [37,38]. Therefore, these results demonstrate that, among the four dietary selenium sources evaluated at 0.5 mg/kg, selenium-enriched yeast is the most effective in enhancing selenium biofortification of pork.

Given that selenium-enriched yeast achieved the highest deposition efficiency in the *longissimus dorsi* (SeY > SeS = SeSP = SeN) at the 0.5 mg/kg supplementation level, we subsequently investigated the underlying transcriptomic changes associated with this superior selenium enrichment. RNA sequencing of the *longissimus dorsi* muscle was performed to compare transcriptomic profiles between the SeY group and the SeS, SeSP, and SeN groups. This yielded 411, 219, and 340 DEGs in the SeS vs. SeY, SeSP vs. SeY, and SeN vs. SeY comparisons, respectively (341/147/204 upregulated; 70/72/136 downregulated). Importantly, 34 DEGs overlapped across all three comparisons, with 27 genes exhibiting consistent upregulation in the SeY group. The upregulation of *S100A1* [39] and *S100A6* [40] (calcium-binding proteins responsive to oxidative and mechanical stress) and *MT1A* [41] (a metallothionein with potent antioxidant and metal-detoxifying capacity) indicates an augmented cellular buffering system against redox perturbations. Concurrently, the increased expression of extracellular matrix-related genes, including *COL22A1* [42] (collagen type XXII at the muscle–tendon junction), *TNC* [43] (tenascin-C, involved in mechanotransduction), and *PRG4* [44] (proteoglycan 4, which possesses anti-inflammatory properties), suggests a reinforcement of muscle structural organization and tissue lubrication, likely contributing to improved meat quality traits such as water-holding capacity. Furthermore, the elevation of immune-regulatory genes (*CD200R1* [45], *FCER1G* [46], *GIMAP4* [47], and *IFI30* [48]) reflects the establishment of an anti-inflammatory microenvironment, while the upregulation of transcription factors *FOSL1* [49] (AP-1 family, stress-responsive) and *E2F8* [50] (cell cycle regulation), along with tissue repair-associated *GPNMB* [51], points to enhanced cellular renewal and regenerative capacity. Additional upregulated genes, including *SCIN* [52] (actin-severing protein), *BAIAP2L1* [53] (cytoskeletal dynamics), *HABP2* [54] (hyaluronan-binding protein), and various proteases (*PRSS55* [55], *PRSS35* [56]) and neuronal/calcium-binding factors (*NECAB3* [57], *UNC13A* [58], *RRH* [59], *GALP* [60]), collectively support extracellular matrix remodeling, cellular plasticity, and neuromuscular signaling adaptations under elevated-selenium conditions. GO enrichment analysis revealed that response to external stimulus and extracellular region were consistently enriched across all three comparisons (SeS_vs_SeY, SeSP_vs_SeY, and SeN_vs_SeY). In addition, KEGG pathway analysis identified viral protein interaction with cytokine and cytokine receptor as a shared enriched pathway among the same comparisons. Collectively, these findings indicate that selenium-enriched yeast outperformed sodium selenite, selenium-enriched seaweed polysaccharide, and nano-selenium in promoting selenium bioaccumulation in the *longissimus dorsi* muscle of pigs. The resultant selenium-enriched muscle tissue exhibited potential beneficial functions mediated primarily through modulation of external stimulus response, extracellular region homeostasis, and cytokine–cytokine receptor crosstalk. Lastly, it was acknowledged that functional analyses of muscle could directly demonstrate the beneficial effects of SeY on muscle function, which is warranted in future research.

## 5. Conclusions

Dietary supplementation with selenium-enriched yeast (SeY) markedly enhanced selenium accumulation in the muscle of Tibetan pigs compared with sodium selenite, seaweed selenium, and nano-selenium at the 0.5 mg/kg supplementation level. Additionally, transcriptome analysis of the *longissimus dorsi* muscle identified 34 differentially expressed genes that were consistently upregulated in the SeY group relative to the other three selenium sources. Functional enrichment analysis further revealed that the SeY group was enriched in the response to external stimulus and extracellular region (GO terms), as well as viral protein interaction with cytokine and cytokine receptor (KEGG pathway).

## Figures and Tables

**Table 1:** Ingredient composition and nutrient level of the basal diet (as-fed basis).

Ingredient	Content	Nutrient	Content ^3^
Corn, %	53.50	Digestible energy, MJ/kg	14.87
Soybean meal, %	17.50	Crude protein, %	15.00
Rice bran, %	16.00	Lysine, %	0.91
Wheat bran, %	10.00	Methionine, %	0.26
Limestone, %	1.50	Calcium, %	0.72
Dicalcium phosphate, %	0.50	Total phosphorus, %	0.66
Salt, %	0.35	Available phosphorus, %	0.25
L-Lysine hydrochloride (98.5%), %	0.20		
Choline chloride (60%)	0.10		
Vitamin premix ^1^, %	0.10		
Mineral premix ^2^, %	0.25		

^1^ Vitamin premix provided the following per kilogram of complete feed: 6500 IU vitamin A, 2000 IU vitamin D_3_, 20 mg vitamin E, 2 mg vitamin K_3_, 2 mg vitamin B_1_, 5 mg vitamin B_2_, 3 mg vitamin B_6_, 20 μg vitamin B_12_, 30 mg niacin, and 16 mg D-pantothenic acid. ^2^ Mineral premix provided the following per kilogram of complete feed: 10 mg copper, 88 mg iron, 72 mg zinc, 40 mg manganese, and 0.24 mg iodine. ^3^ Calculated values.

**Table 2:** Effects of dietary selenium sources on the growth and slaughter performance of Tibetan pigs.

Items	SeS	SeY	SeSP	SeN	SEM	*p*-Value
Average daily feed intake (kg)	2.03	1.96	1.93	1.83	0.033	0.22
Average daily gain (kg)	0.43	0.49	0.47	0.44	0.02	0.64
Feed conversion ratio	4.50	4.07	4.17	4.04	0.43	0.46
Carcass weight (kg)	51.24	51.91	50.09	50.18	1.42	0.98
Body length (cm)	88.83	85.40	85.86	83.83	0.83	0.20
Body diagonal length (cm)	65.50	63.20	63.71	61.83	0.92	0.60
Thickness of backfat (cm)	2.70	2.72	2.68	2.25	0.15	0.99
*Longissimus dorsi* eye area (cm^2^)	19.02	19.63	20.33	17.08	0.65	0.50

Data are presented as means and pooled SEM (*n* = 6). Abbreviations: SeS, sodium selenite; SeY, selenium-enriched yeast; SeSP, selenium-enriched seaweed polysaccharide; SeN, nano-selenium.

**Table 3:** Effects of dietary selenium sources on meat (*longissimus dorsi*) quality of Tibetan pigs.

Item	SeS	SeY	SeSP	SeN	SEM	*p*-Value
Moisture, %	72.95	72.48	72.34	72.68	0.24	0.84
Crude protein, %	19.78	20.05	19.77	19.07	0.27	0.66
Crude fat, %	5.23	5.76	5.79	6.13	0.20	0.49
Crude ash, %	1.54	1.30	1.64	1.50	0.06	0.26
pH	6.26	6.20	6.10	6.06	0.06	0.59
Lightness (L*)	37.41	35.75	38.82	37.43	0.45	0.11
Redness (a*)	2.32	2.18	0.99	2.09	0.24	0.23
Yellowness (b*)	5.33	5.31	6.04	5.88	0.17	0.33
Shear force, N	82.54	79.50	77.03	69.23	2.31	0.07
Cooking loss, %	20.26	20.14	18.06	21.83	0.82	0.46
Drip loss, %	2.38 ^b^	2.13 ^b^	3.07 ^b^	5.04 ^a^	0.39	0.02

^a,b^ Values in the same row with different superscripts are significantly different (*p* < 0.05). Data are presented as means and SEM (*n* = 6). Abbreviations: SeS, sodium selenite; SeY, selenium-enriched yeast; SeSP, selenium-enriched seaweed polysaccharide; SeN, nano-selenium.

**Table 4:** Overlap differentially expressed genes (DEGs) in *longissimus dorsi* for SeS vs. SeY, SeSP vs. SeY, and SeN vs. SeY.

Gene Name	Description	SeS_vs_SeY		SeSP_vs_SeY		SeN_vs_SeY	
		Log_2_^(Fold Change)^	*p*-Value	Log_2_^(Fold Change)^	*p*-Value	Log_2_^(Fold Change)^	*p*-Value
*S100A1*	S100 calcium binding protein A1	1.13	6.77 × 10^−3^	1.08	0.022	1.85	6.25 × 10^−7^
*S100A6*	S100 calcium binding protein A6	1.16	5.45 × 10^−3^	1.01	4.60 × 10^−3^	1.47	4.68 × 10^−5^
*COL22A1*	Collagen type XXII alpha 1 chain	2.37	2.25 × 10^−3^	1.97	0.013	3.82	1.30 × 10^−5^
*TNC*	Tenascin C	2.78	1.11 × 10^−10^	1.64	6.12 × 10^−4^	1.97	3.16 × 10^−4^
*PRG4*	Proteoglycan 4	3.15	0.015	3.87	2.45 × 10^−4^	4.57	4.32 × 10^−4^
*MT1A*	Metallothionein 1A	3.15	1.13 × 10^−3^	3.47	2.53 × 10^−4^	3.75	6.16 × 10^−4^
*GPNMB*	Glycoprotein nmb	1.46	1.31 × 10^−3^	1.10	0.019	1.80	6.96 × 10^−4^
*GIMAP4*	GTPase, IMAP family member 4	1.77	4.31 × 10^−3^	1.37	0.019	1.74	7.52 × 10^−4^
*CD200R1*	CD200 receptor 1	1.57	3.41 × 10^−3^	1.14	0.031	1.67	2.26 × 10^−3^
*FCER1G*	Fc fragment of IgE receptor Ig	1.69	6.46 × 10^−4^	1.09	0.033	1.60	3.31 × 10^−3^
*SCIN*	scinderin	1.55	0.012	1.70	0.014	1.90	3.63 × 10^−3^
*FOSL1*	FOS-like 1, AP-1 transcription factor subunit	2.53	7.13 × 10^−4^	1.87	0.020	2.41	3.96 × 10^−3^
*BAIAP2L1*	BAI1 associated protein 2 like 1	3.62	6.97 × 10^−5^	2.09	4.01 × 10^−3^	2.12	5.42 × 10^−3^
*HABP2*	Hyaluronan binding protein 2	1.07	0.021	1.26	0.039	1.59	7.51 × 10^−3^
*PRSS55*	Serine protease 55	1.50	0.041	1.39	0.047	2.05	7.78 × 10^−3^
*GALP*	Galanin-like peptide	2.53	8.45 × 10^−3^	3.56	7.87 × 10^−0.4^	2.84	9.34 × 10^−3^
*IFI30*	IFI30 lysosomal thiol reductase	1.17	3.40 × 10^−3^	1.07	4.72 × 10^−0.3^	1.21	0.011
*NECAB3*	N-terminal EF-hand calcium binding protein 3	1.74	6.03 × 10^−3^	1.47	0.016	1.59	0.014
*PRSS35*	Serine protease 35	4.51	1.18 × 10^−3^	3.07	0.020	3.26	0.017
*UNC13A*	Unc-13 homolog A	2.72	0.016	2.13	0.038	2.49	0.020
*E2F8*	E2F transcription factor 8	2.04	0.020	1.94	0.011	1.99	0.032
*RRH*	Retinal pigment epithelium-derived rhodopsin homolog	1.71	0.027	1.99	0.015	1.69	0.042
*LOC100512920*	Intercellular adhesion molecule 5	2.46	9.15 × 10^−3^	2.44	7.25 × 10^−3^	3.21	1.78 × 10^−3^
*LOC110258709*	C-type lectin domain family 4 member A-like	3.24	1.61 × 10^−3^	2.07	0.025	2.76	7.17 × 10^−3^
*LOC110261486*	-	1.83	0.022	1.92	1.27 × 10^−0.3^	1.53	0.022
*LOC100516039*	C-C motif chemokine 23	2.50	8.23 × 10^−4^	1.88	8.39 × 10^−0.3^	1.89	0.022
*LOC100521785*	Zinc finger protein 2	2.14	0.034	2.13	0.045	2.68	0.025
*RETSAT*	Retinol saturase	−1.12	2.33 × 10^−7^	−1.09	2.58 × 10^−5^	−1.60	7.54 × 10^−6^
*PTCHD4*	Patched domain containing 4	−3.87	6.55 × 10^−3^	−2.90	0.036	−3.05	6.24 × 10^−4^
*NFATC3*	Nuclear factor of activated T cells 3	−1.23	2.92 × 10^−6^	−1.06	8.89 × 10^−4^	−1.13	1.86 × 10^−3^
*CBL*	Cbl proto-oncogene	−1.67	4.27 × 10^−3^	−1.56	9.05 × 10^−3^	−1.46	4.93 × 10^−3^
*KCNJ3*	Potassium voltage-gated channel subfamily J member 3	−1.17	1.03 × 10^−3^	−1.10	4.99 × 10^−0.5^	−1.14	7.03 × 10^−3^
*PAIP2B*	-	−1.47	6.25 × 10^−10^	−1.27	7.07 × 10^−6^	−1.44	2.77 × 10^−6^
*LOC102161970*	-	−1.42	0.012	−1.12	0.029	−1.30	0.020

Abbreviations: SeS, sodium selenite; SeY, selenium-enriched yeast; SeSP, selenium-enriched seaweed polysaccharide; SeN, nano-selenium.

## Data Availability

The original contributions presented in this study are included in the article/Supplementary Materials. Further inquiries can be directed to the corresponding authors.

## Supplementary Materials

The following supporting information can be downloaded at: https://www.mdpi.com/article/10.3390/foods15183202/s1, File S1: Differentially expressed genes (DEGs) identification for SeS_vs_SeY; File S2: Differentially expressed genes (DEGs) identification for SeSP_vs_SeY; File S3: Differentially expressed genes (DEGs) identification for SeN_vs_SeY; File S4: Statistics of GO enrichment items for SeS_vs_SeY; File S5: Statistics of GO enrichment items for SeSP_vs_SeY; File S6: Statistics of GO enrichment items for SeN_vs_SeY; File S7: Statistics of KEGG enrichment pathways for SeS_vs_SeY; File S8: Statistics of KEGG enrichment pathways for SeSP_vs_SeY; File S9: Statistics of KEGG enrichment pathways for SeN_vs_SeY.

## References

[1] Pecoraro B.M., Leal D.F., Frias-De-Diego A., Browning M., Odle J., Crisci E. (2022). The health benefits of selenium in food animals: A review. J. Anim. Sci. Biotechnol..

[2] Dinh Q.T., Cui Z., Huang J., Tran T.A.T., Wang D., Yang W., Zhou F., Wang M., Yu D., Liang D. (2018). Selenium distribution in the Chinese environment and its relationship with human health: A review. Environ. Int..

[3] Finley J.W. (2007). Increased intakes of selenium-enriched foods may benefit human health. J. Sci. Food Agric..

[4] Wan J., Zhang M., Adhikari B. (2018). Advances in selenium-enriched foods: From the farm to the fork. Trends Food Sci. Technol..

[5] Qiu X., Lu P., Xiao M., Zeng S., Li L., Yu H., Deng A., Zhu M., Xu E., Zeng X. (2026). Se-enriched yeast improves meat quality through glycerophospholipid metabolism in finishing pigs: Insights from a multi-omics analysis. J. Anim. Sci. Biotechnol..

[6] National Research Council (NRC) (2012). Nutrient Requirements of Swine.

[7] (2020). Nutrient Requirements of Swine.

[8] ARC (1981). The Nutrient Requirements of Pigs.

[9] Ju Y., Liu M., Huang L., Luo Y., Qi L., Ye J., Wu X., Cao N., Bo J., Liu X. (2021). Effects of selenium *Auricularia cornea* culture supplementation on growth performance, antioxidant status, tissue selenium concentration and meat quality in growing-finishing pigs. Animals.

[10] Chen J., Tian M., Guan W., Wen T., Yang F., Chen F., Zhang S., Song J., Ren C., Zhang Y. (2019). Increasing selenium supplementation to a moderately-reduced energy and protein diet improves antioxidant status and meat quality without affecting growth performance in finishing pigs. J. Trace Elem. Med. Biol..

[11] Danso O.P., Asante-Badu B., Zhang Z., Song J., Wang Z., Yin X., Zhu R. (2023). Selenium biofortification: Strategies, progress and challenges. Agriculture.

[12] Kieliszek M., Bierla K., Jiménez-Lamana J., Kot A.M., Alcántara-Durán J., Piwowarek K., Błażejak S., Szpunar J. (2020). Metabolic response of the yeast *Candida utilis* during enrichment in selenium. Int. J. Mol. Sci..

[13] Zhou W., Yang Z., Han J., Chen X., Zou T., You J., Chen J. (2024). An updated review of emerging sources of selenium in weaned piglet nutrition. Animals.

[14] Chen J., Han J.H., Guan W.T., Chen F., Wang C.X., Zhang Y.Z., Lv Y.T., Lin G. (2016). Selenium and vitamin E in sow diets: I. Effect on antioxidant status and reproductive performance in multiparous sows. Anim. Feed. Sci. Technol..

[15] Zhong H., Huang Z., Li L., Chen X., Zou T., Chen J., You J. (2024). Selenomethionine supplementation mitigates liver dysfunction, oxidative injury and apoptosis through enhancing antioxidant capacity and inhibiting JNK MAPK pathway in piglets fed deoxynivalenol-contaminated diets. Antioxidants.

[16] Huang Z., Zhong H., Li T., Wang Z., Chen X., Zou T., You J., Chen J. (2024). Selenomethionine alleviates deoxynivalenol-induced oxidative injury in porcine intestinal epithelial cells independent of MAPK pathway regulation. Antioxidants.

[17] Gorska S., Maksymiuk A., Turło J. (2021). Selenium-containing polysaccharides—Structural diversity, biosynthesis, chemical modifications and biological activity. Appl. Sci..

[18] Ponton D.E., Graves S.D., Fortin C., Janz D., Amyot M., Schiavon M. (2020). Selenium interactions with algae: Chemical processes at biological uptake sites, bioaccumulation, and intracellular metabolism. Plants.

[19] Wang S., Tang C., Li J., Wang Z., Meng F., Luo G., Xin H., Zhong J., Wang Y., Li B. (2022). The effects of dietary inclusion of mulberry leaf powder on growth performance, carcass traits and meat quality of Tibetan pigs. Animals.

[20] Yang L., Tao A., Hu H., Ding M., Chen J., Li X., Chen X., Zou T., You J. (2026). Improving meat quality and lipid metabolism of finishing pigs by replacing dietary soybean meal with enzyme–bacteria co-fermented rapeseed meal. Foods.

[21] (2004). Technical Regulation for Testing of Carcass Traits in Lean-Type Pig.

[22] (2016). Determination of Moisture in Foods.

[23] (2016). Determination of Protein in Foods.

[24] (2016). Determination of Fat in Food.

[25] (2016). Determination of Ash in Food.

[26] Zhang J., Zhang C., Meng S., Wang H., Liu D., Guo L., Miao Z. (2025). Evaluation of the effects of acorns on the meat quality and transcriptome profile of finishing Yuxi pigs. Animals.

[27] Tian J., Yun M., Ju W., Long H., Kim J., Kil D.Y., Chang J., Cho S., Kim Y., Han I.K. (2006). Effects of dietary selenium supplementation on growth performance, selenium retention in tissues and nutrient digestibility in growing-finishing pigs. Asian-Australas. J. Anim. Sci..

[28] Zhan X., Wang M., Zhao R., Li W., Xu Z. (2007). Effects of different selenium source on selenium distribution, loin quality and antioxidant status in finishing pigs. Anim. Feed. Sci. Technol..

[29] Gjerlaug-Enger E., Haug A., Gaarder M., Ljøkjel K., Stenseth R.S., Sigfridson K., Egelandsdal B., Saarem K., Berg P. (2015). Pig feeds rich in rapeseed products and organic selenium increased omega-3 fatty acids and selenium in pork meat and backfat. Food Sci. Nutr..

[30] Jing J., Wang J., Wu Q., Yin S., He Z., Tang J., Jia G., Liu G., Chen X., Tian G. (2024). Nano-Se exhibits limited protective effect against heat stress induced poor breast muscle meat quality of broilers compared with other selenium sources. J. Anim. Sci. Biotechnol..

[31] Mahan D., Cline T., Richert B. (1999). Effects of dietary levels of selenium-enriched yeast and sodium selenite as selenium sources fed to growing-finishing pigs on performance, tissue selenium, serum glutathione peroxidase activity, carcass characteristics, and loin quality. J. Anim. Sci..

[32] Zhang K., Zhao Q., Zhan T., Han Y., Tang C., Zhang J. (2020). Effect of different selenium sources on growth performance, tissue selenium content, meat quality, and selenoprotein gene expression in finishing pigs. Biol. Trace Elem. Res..

[33] Jiang J., Tang X., Xue Y., Lin G., Xiong Y.L. (2017). Dietary linseed oil supplemented with organic selenium improved the fatty acid nutritional profile, muscular selenium deposition, water retention, and tenderness of fresh pork. Meat Sci..

[34] Chen J., Xing Y., Nie M., Xu M., Huang H., Xie H., Liao J., Lin X., Duan J., Zhang J. (2024). Comparative effects of various dietary selenium sources on growth performance, meat quality, essential trace elements content, and antioxidant capacity in broilers. Poult. Sci..

[35] Nickel A., Kottra G., Schmidt G., Danier J., Hofmann T., Daniel H. (2009). Characteristics of transport of selenoamino acids by epithelial amino acid transporters. Chem. Biol. Interact..

[36] Thiry C., Ruttens A., Pussemier L., Schneider Y.J. (2013). An in vitro investigation of species-dependent intestinal transport of selenium and the impact of this process on selenium bioavailability. Br. J. Nutr..

[37] Kieliszek M., Błażejak S., Gientka I., Bzducha-Wróbel A. (2015). Accumulation and metabolism of selenium by yeast cells. Appl. Microbiol. Biotechnol..

[38] Bano I., Jeevanandam J., Tsenov G. (2025). In vitro study of selenomethionine’s concentration-dependent effects in a 6-OHDA model of Parkinson’s disease. In Vitro Model.

[39] Prosser B.L., Hernández-Ochoa E.O., Lovering R.M., Andronache Z., Zimmer D.B., Melzer W., Schneider M.F. (2010). S100A1 promotes action potential-initiated calcium release flux and force production in skeletal muscle. Am. J. Physiol. Cell Physiol..

[40] Wang Y., Kang X., Kang X., Yang F. (2023). S100A6: Molecular function and biomarker role. Biomark. Res..

[41] Yang R., Roshani D., Gao B., Li P., Shang N. (2024). Metallothionein: A comprehensive review of its classification, structure, biological functions, and applications. Antioxidants.

[42] Charvet B., Guiraud A., Malbouyres M., Zwolanek D., Guillon E., Bretaud S., Monnot C., Schulze J., Bader H.L., Allard B. (2013). Knockdown of col22a1 gene in zebrafish induces a muscular dystrophy by disruption of the myotendinous junction. Development.

[43] Imanaka-Yoshida K., Aoki H. (2014). Tenascin-C and mechanotransduction in the development and diseases of cardiovascular system. Front. Physiol..

[44] Alquraini A., Garguilo S., D’Souza G., Zhang L.X., Schmidt T.A., Jay G.D., Elsaid K.A. (2015). The interaction of lubricin/proteoglycan 4 (PRG4) with toll-like receptors 2 and 4: An anti-inflammatory role of PRG4 in synovial fluid. Arthritis Res. Ther..

[45] Linley H., Ogden A., Jaigirdar S., Buckingham L., Cox J., Priestley M., Saunders A. (2023). CD200R1 promotes interleukin-17 production by group 3 innate lymphoid cells by enhancing signal transducer and activator of transcription 3 activation. Mucosal Immunol..

[46] Zhang Y., Wang J., He W., Liu T. (2026). FCER1G: A multifunctional regulator in the immune microenvironment. Exp. Ther. Med..

[47] Heinonen M.T., Kanduri K., Lähdesmäki H.J., Lahesmaa R., Henttinen T.A. (2015). Tubulin-and actin-associating GIMAP4 is required for IFN-γ secretion during Th cell differentiation. Immunol. Cell Biol..

[48] Zhu C., Chen X., Guan G., Zou C., Guo Q., Cheng P., Cheng W., Wu A. (2020). IFI30 is a novel immune-related target with predicting value of prognosis and treatment response in glioblastoma. OncoTargets Ther..

[49] Al-Khayyat W., Laframboise T., Dougherty J., Mendonca M.S., Boreham D.R., Tai T., Thome C., Tharmalingam S. (2025). FRA1 (FOSL1) suppresses neoplastic transformation and modulates radiation responses via transcriptional control of mitogenic and stress-responsive networks. Front. Cell Dev. Biol..

[50] Christensen J., Cloos P., Toftegaard U., Klinkenberg D., Bracken A.P., Trinh E., Heeran M., Di Stefano L., Helin K. (2005). Characterization of E2F8, a novel E2F-like cell-cycle regulated repressor of E2F-activated transcription. Nucleic Acids Res..

[51] Chen Y.F., Lee C.W., Li Y.S.J., Lin W.T., Chen H.Y., Chen Y.C., Lin C.H., Ho J.H.C., Lu L.F., Chien S. (2025). Temporal single-cell sequencing analysis reveals that GPNMB-expressing macrophages potentiate muscle regeneration. Exp. Mol. Med..

[52] Trifaro J., Rose S., Marcu M. (2000). Scinderin, a Ca^2+^-dependent actin filament severing protein that controls cortical actin network dynamics during secretion. Neurochem. Res..

[53] Hadebe S., Savulescu A.F., Khumalo J., Jones K., Mangali S., Mthembu N., Musaigwa F., Maepa W., Ndlovu H., Ngomti A. (2025). Immunoglobulin M regulates airway hyperresponsiveness independent of T helper 2 allergic inflammation. eLife.

[54] Mambetsariev N., Mirzapoiazova T., Mambetsariev B., Sammani S., Lennon F.E., Garcia J.G., Singleton P.A. (2010). Hyaluronic acid binding protein 2 is a novel regulator of vascular integrity. Arterioscler. Thromb. Vasc. Biol..

[55] Zhu F., Li W., Zhou X., Chen X., Zheng M., Cui Y., Liu X., Guo X., Zhu H. (2021). PRSS55 plays an important role in the structural differentiation and energy metabolism of sperm and is required for male fertility in mice. J. Cell Mol. Med..

[56] Sänger C.S., Cernakova M., Wietecha M.S., Garau Paganella L., Labouesse C., Dudaryeva O.Y., Roubaty C., Stumpe M., Mazza E., Tibbitt M.W. (2023). Serine protease 35 regulates the fibroblast matrisome in response to hyperosmotic stress. Sci. Adv..

[57] Bueno D., Schäfer M.K., Wang S., Schmeisser M.J., Methner A. (2025). NECAB family of neuronal calcium-binding proteins in health and disease. Neural Regen. Res..

[58] Willemse S.W., Harley P., van Eijk R.P.A., Demaegd K.C., Zelina P., Pasterkamp R.J., van Damme P., Ingre C., van Rheenen W., Veldink J.H. (2023). UNC13A in amyotrophic lateral sclerosis: From genetic association to therapeutic target. J. Neurol. Neurosurg. Psychiatry.

[59] Leung C., Guitard J.J., Senay C., Bourret A., Chabot D., Parent G.J. (2025). Past environments modulate response to fluctuating temperatures in a marine fish species, Sebastes fasciatus. J. Exp. Biol..

[60] Robinson J., Bartfai T., Langel U. (2006). Galanin/GALP receptors and CNS homeostatic processes. CNS Neurol. Disord. Drug Targets.

